# Activity of Phage–Lactoferrin Mixture against Multi Drug Resistant *Staphylococcus aureus* Biofilms

**DOI:** 10.3390/antibiotics11091256

**Published:** 2022-09-16

**Authors:** Katarzyna Kosznik-Kwaśnicka, Natalia Kaźmierczak, Lidia Piechowicz

**Affiliations:** 1 Department of Medical Microbiology, Faculty of Medicine, Medical University of Gdańsk, Dębowa 25, 80-204 Gdansk, Poland; 2 Laboratory of Phage Therapy, Institute of Biochemistry and Biophysics, Polish Academy of Sciences, Kładki 24, 80-822 Gdansk, Poland

**Keywords:** phage therapy, phages, lactoferrin, biofilm, *Staphylococcus aureus*, MDRSA, MRSA

## Abstract

Biofilms are complex bacterial structures composed of bacterial cells embedded in extracellular polymeric substances (EPS) consisting of polysaccharides, proteins and lipids. As a result, biofilms are difficult to eradicate using both mechanical methods, i.e., scraping, and chemical methods such as disinfectants or antibiotics. Bacteriophages are shown to be able to act as anti-biofilm agents, with the ability to penetrate through the matrix and reach the bacterial cells. However, they also seem to have their limitations. After several hours of treatment with phages, the biofilm tends to grow back and phage-resistant bacteria emerge. Therefore, it is now recommended to use a mixture of phages and other antibacterial agents in order to increase treatment efficiency. In our work we have paired staphylococcal phages with lactoferrin, a protein with proven anti-biofilm proprieties. By analyzing the biofilm biomass and metabolic activity, we have observed that the addition of lactoferrin to phage lysate accelerated the anti-biofilm effect of phages and also prevented biofilm re-growth. Therefore, this combination might have a potential use in biofilm eradication procedures in medical settings.

## 1. Introduction

*Staphylococcus aureus* is a common nosocomial pathogen that can be responsible for wound infections, hospital-acquired pneumonia or sepsis [1,2]. The emergence of antibiotic resistance, especially to methicillin among nosocomial strains, resulted in difficulties in treatment, which is then responsible for prolonged hospital stays, increased mortality and morbidity of infections [3]. The rates of methicillin resistance among clinical isolates vary from country to country, ranging from a small percent in Scandinavian countries to over 50% in the U.S. and Asian countries [3,4,5]. *S. aureus* can form a biofilm—a complex bacterial structure composed of bacterial cells embedded in extracellular polymeric substance (EPS) that can attach to both organic and inorganic surfaces [6]. The ability to form a biofilm plays an important role in *S. aureus* virulence as cells in biofilms are more resistant to various eradication mechanisms. Furthermore, individual cells can detach from the original biofilm and establish new sites of infection or mediate an acute infection such as sepsis [7]. Bacteriophages, the viruses that infect bacteria, have been shown to be able to successfully eradicate biofilms [8,9]. Phages can prevent biofilm formation and maturation by destroying bacteria in the outer layer of biofilm and planktonic cells. They can also penetrate existing biofilms and eliminate the biofilm structure as phage lytic enzymes, depolymerase and lysins, are being released from the cells upon phage progeny release [10]. However, even phages have their limitations. In some cases, the re-growth of biofilm was observed, and the emergence of resistant bacteria was reported [11,12]. Therefore, it is recommended to pair the phages with other antimicrobials [13,14]. Since the global consensus is to reduce the use of antibiotics, other compounds with antimicrobial activity are also being researched. Lactoferrin is an 80 kDa protein of the transferrin family of non-heme, iron-binding glycoproteins and an important part of the innate immune system. It is present in the blood, at the mucosa, and it is secreted with fluids such as milk, tears, sweat or semen [15]. It has been shown that lactoferrin can act as anti-biofilm agent reducing the biomass, loosening the biofilm structure and enabling its dispersion [15,16,17]. The detailed mechanisms of lactoferrin’s anti-biofilm activity remain to be discovered [15]. However, its potential in treatment should be investigated. Therefore, we have decided to test the combination of phages and lactoferrin against clinical strains of multidrug-resistant *S. aureus* (MDRSA). We have discovered that the phage–lactoferrin mixture significantly reduced biofilm metabolic activity and biomass. Furthermore, the addition of lactoferrin to phage lysate slowed down the process of biofilm re-growth. We believe that combined phage–lactoferrin treatment could be implemented in the eradication of biofilms formed by nosocomial pathogens and should be studied further to fully evaluate its potential.

## 2. Results

### 2.1. Lactoferrin Influence on Phage Activity

**Figure 1:**
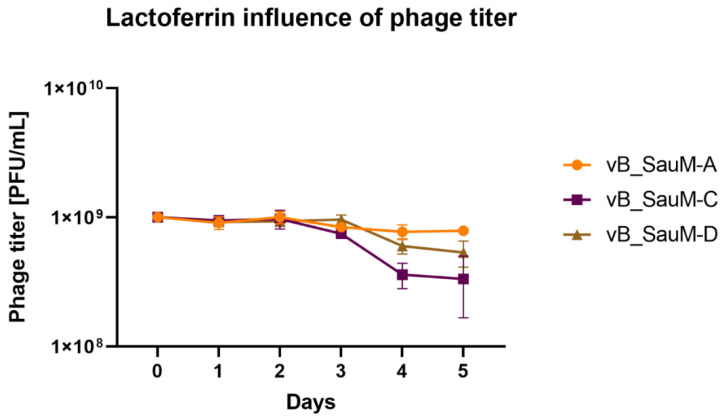
Influence of lactoferrin (10 mg/mL) on phage titer during storage period. Arithmetic mean of triplicates, with error bars representing SD.

Bacteriophages vB_SenM-A, vB_SauM-C and vB_SauM-D have previously been characterized and have proven activity against MDRSA biofilms when used alone [8,18]. In order to assess if phage and lactoferrin can be used simultaneously in the form of a cocktail, the phage–lactoferrin mixture was stored at 4 °C for a period of 5 days, with titration performed every 24 h. The phage titer began to drop after 3 days of incubation in case of phages vB_SauM-C and vB_SauM-D (Figure 1). The highest drop in activity (assessed using Plaque Forming Unit—PFU/mL) was observed for phage vB_SauM-C. The titer dropped from an initial 10^9^ PFU/mL to 6 × 10^8^ PFU/mL after 4 days of incubation.

### 2.2. Lactoferrin Influence on Biofilm Formed by MDRSA Strains

**Figure 2:**
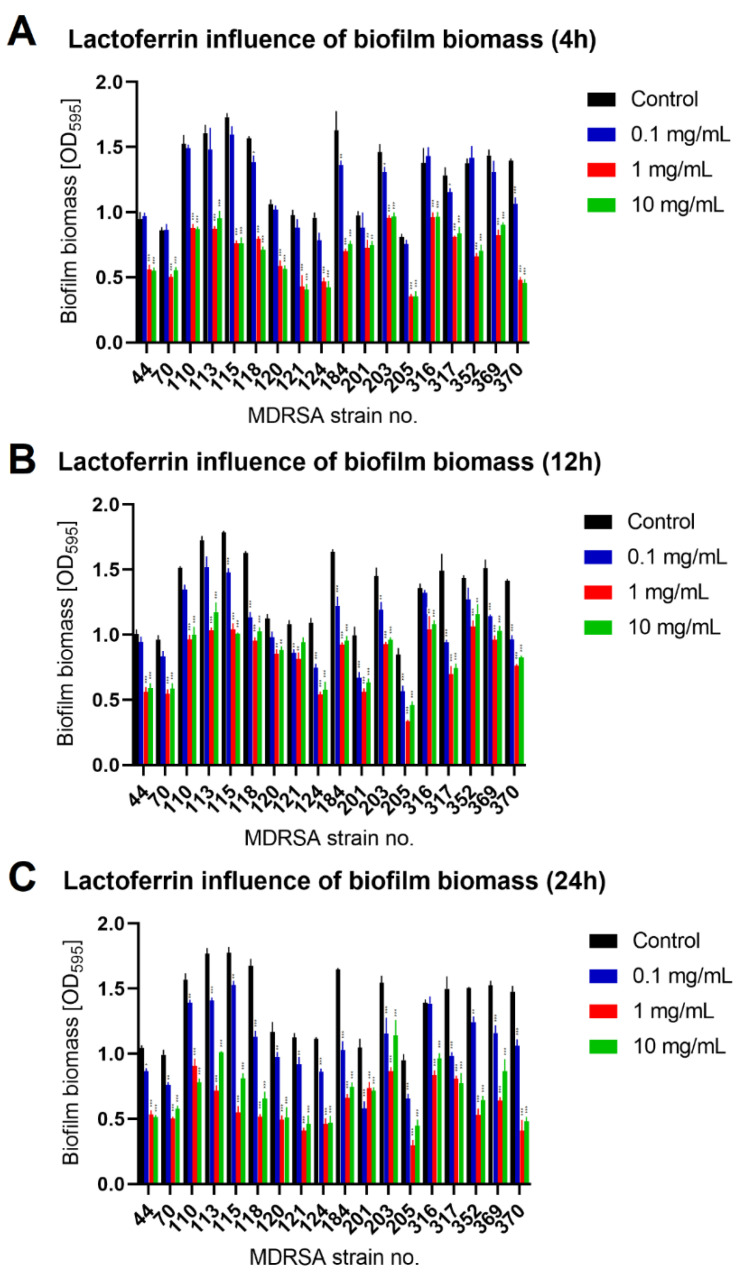
Influence of lactoferrin (0.1 mg/mL, 1.0 mg/mL and 10 mg/mL) on MDRSA biofilm biomass after 4 h (**A**), 12 h (**B**) and 24 h (**C**) of incubation. Mean of triplicates, with error bars representing SD. Statistical analysis was performed using *t*-test, *p* < 0.05 (*), *p* < 0.01 (**), *p* < 0.001 (***).

**Figure 3:**
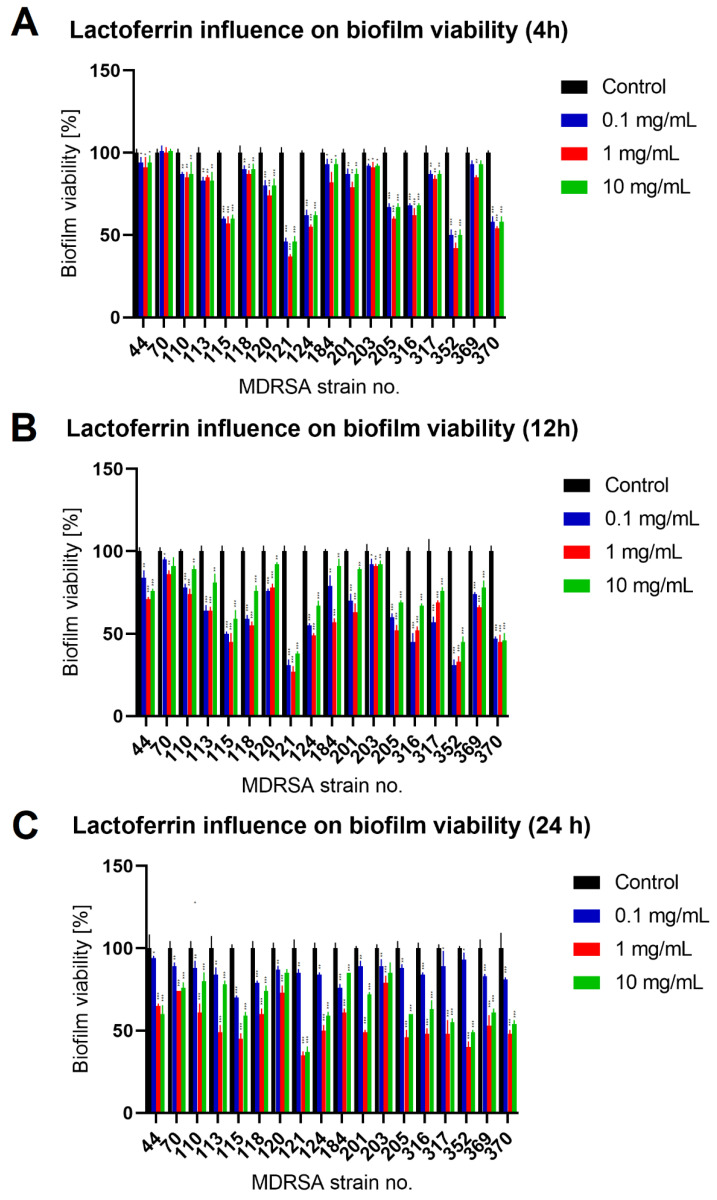
Influence of lactoferrin (0.1 mg/mL, 1.0 mg/mL and 10 mg/mL) on MDRSA biofilm viability after 4 h (**A**), 12 h (**B**) and 24 h (**C**) of incubation. Mean of triplicates, with error bars representing SD. Statistical analysis was performed using *t*-test, *p* < 0.05 (*), *p* < 0.01 (**), *p* < 0.001 (***).

To evaluate lactoferrin anti-biofilm activity, we selected appropriate MDRSA strains that were classified as strong biofilm producers during our previous studies [19]. Mature MDRSA biofilms treated with three different concentrations of lactoferrin: 0.1 mg/mL, 1.0 mg/mL and 10 mg/mL, which were chosen based on literature data [16,17]. After 4 h, 12 h of 24 h of incubation with lactoferrin total biofilm biomass (Crystal Violet staining), biofilm metabolic activity (MTT-Formazan assay) and the colony-forming unit (CFU/mL) numbers were assessed. It was found that lactoferrin significantly decreased biofilm biomass and viability in concentrations of 1.0 mg/mL and 10 mg/mL after 12 h and 24 h of incubation, respectively (Figure 2 and Figure 3). In most cases, statistical analysis revealed no difference between 1.0 mg/mL and 10 mg/mL concentrations of lactoferrin on biofilm biomass and viability.

**Figure 4:**
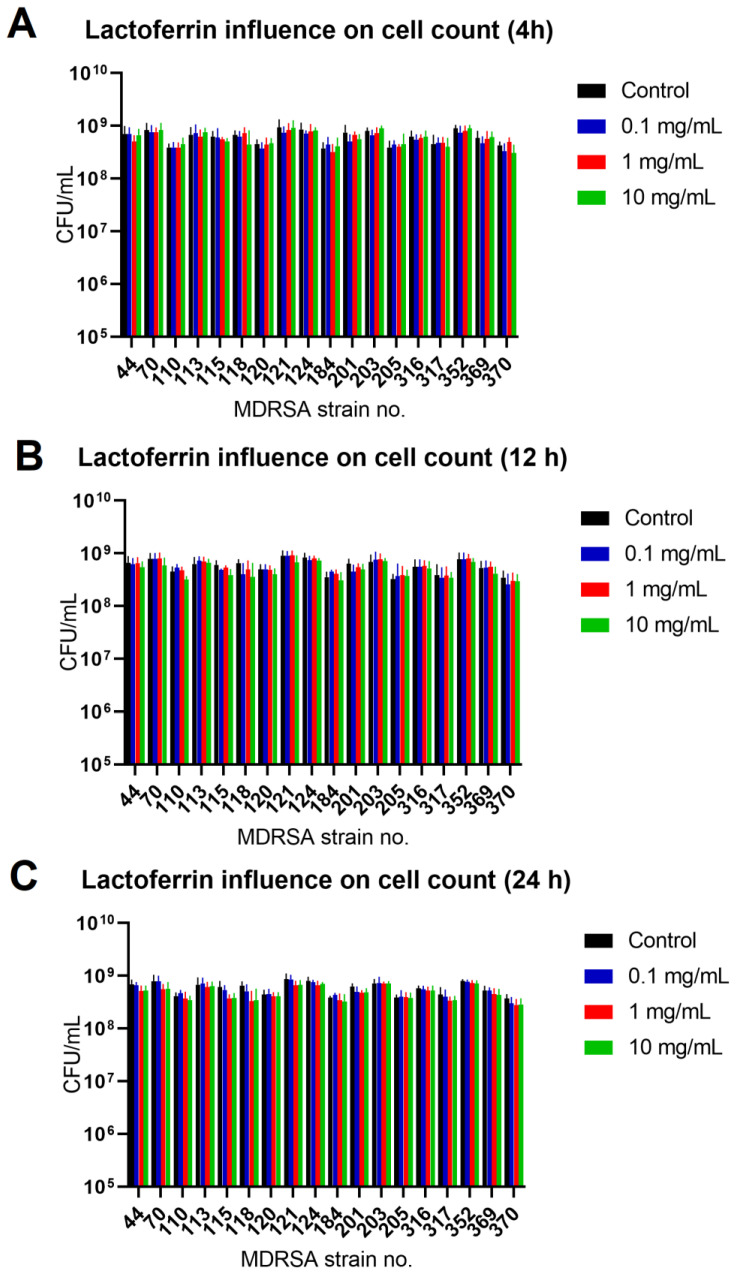
Influence of lactoferrin (0.1 mg/mL, 1.0 mg/mL and 10 mg/mL) on MDRSA biofilm CFU/mL count after 4 h (**A**), 12 h (**B**) and 24 h (**C**) of incubation. Mean of triplicates, with error bars representing SD. Statistical analysis performed using *t*-test showed no significance.

The effect was most pronounced for MDRSA strain no. 121 (more that 50% reduction in both biofilm biomass and viability) (Figure 2 and Figure 3). While strains no. 70, 120 and 203 were the least influenced in case of viability (Figure 3), significant reduction in biofilm biomass was observed (Figure 2). We did not, however, observe a significant reduction in the number of cells creating the biofilm (Figure 4).

### 2.3. Biofilm Eradication by Phage–Lactoferrin Mixture

In order to assess if lactoferrin will influence phage anti-biofilm activity, a phage–lactoferrin mixture was prepared. The mixture consisted of one of three previously characterized phages (vB_SauM-A, vB_SauM-C and vB_SauM-D) with proven activity against MDRSA strains [8,18]. Based on our previous studies and presented data on lactoferrin activity, we have chosen the concentration of phage to be 10^9^ PFU/mL and 1.0 mg/mL for lactoferrin.

**Figure 5:**
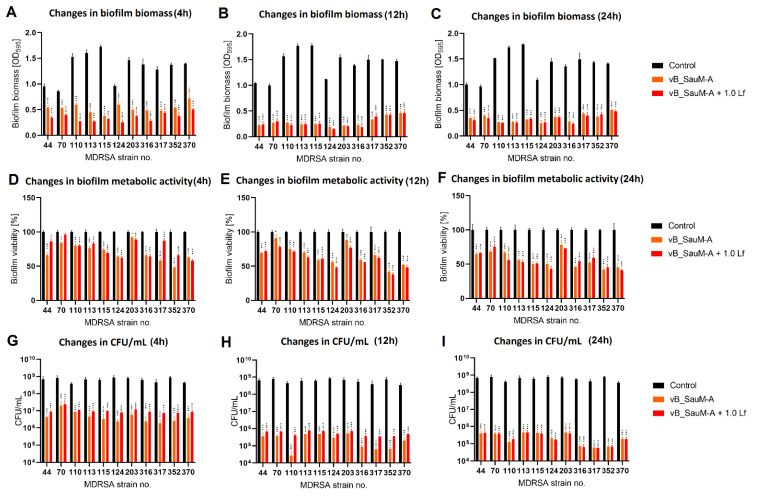
Influence of phage vB_SauM-A and vB_SauM-A+ 1.0 mg/mL lactoferrin (Lf) on MDRSA biofilm: biomass (**A**–**C**), metabolic activity (**D**–**F**) and CFU/mL count (**G**–**I**) after 4 h (**A**,**D**,**G**), 12 h (**B**,**E**,**H**) and 24 h (**C**,**F**,**I**) of incubation. Mean of triplicates, with error bars representing SD. Statistical analysis was performed using *t*-test, *p* < 0.05 (*), *p* < 0.01 (**), *p* < 0.001 (***).

**Figure 6:**
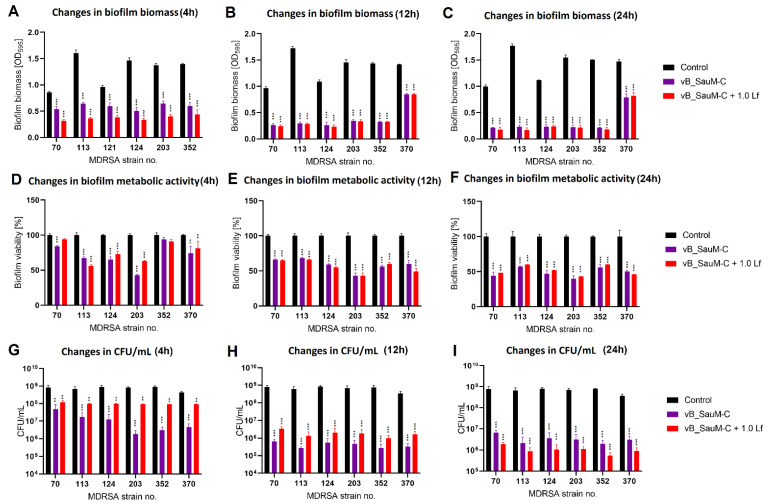
Influence of phage vB_SauM-C and vB_SauM-C+ 1.0 mg/mL lactoferrin (Lf) on MDRSA biofilm: biomass (**A**–**C**), metabolic activity (**D**–**F**) and CFU/mL count (**G**–**I**) after 4 h (**A**,**D**,**G**), 12 h (**B**,**E**,**H**) and 24 h (**C**,**F**,**I**) of incubation. Mean of triplicates, with error bars representing SD. Statistical analysis was performed using *t*-test, *p* < 0.01 (**), *p* < 0.001 (***).

**Figure 7:**
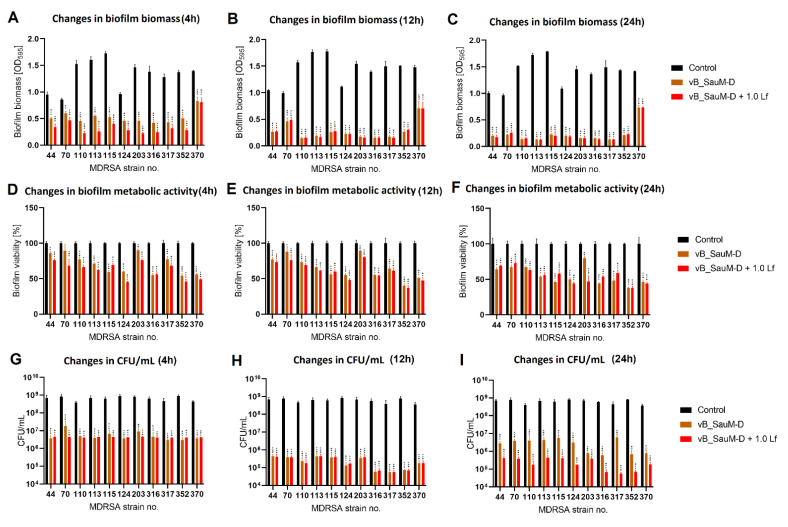
Influence of phage vB_SauM-D and vB_SauM-D+ 1.0 mg/mL lactoferrin (Lf) on MDRSA biofilm: biomass (**A**–**C**), metabolic activity (**D**–**F**) and CFU/mL count (**G**–**I**) after 4 h (**A**,**D**,**G**), 12 h (**B**,**E**,**H**) and 24 h (**C**,**F**,**I**) of incubation. Mean of triplicates, with error bars representing SD. Statistical analysis was performed using *t*-test, *p* < 0.05 (*), *p* < 0.01 (**), *p* < 0.001 (***).

We have observed that the phage–lactoferrin mixture was more efficient against bacterial biofilm in the first hours after administration. The reduction was especially visible in biofilm biomass and metabolic activity for phage vB_SauM-A (Figure 5). In the case of phage vB_SauM-C, we have only observed an increased reduction in biofilm biomass after the first four hours of incubation (Figure 6A). Biofilm biomass and metabolic activity reduction for phage vB_SauM-D (Figure 7) was similar to phage vB_SauM-A. After 12 h, the effectiveness of the phage–lactoferrin cocktail and phage lysate equaled out, and statistical analysis revealed no significant differences. However, after 24 h of incubation in the case of phages vB_SauM-C and vB_SauM-D, we have observed that the CFU/mL would start to increase, signaling the re-growth of biofilm and possible emergence of resistant bacteria (Figure 5I and Figure 7I). When lactoferrin was added to the mixture, this effect was not observed, and in the case of phage vB_SauM-D, there was a statistically significant difference between the phage lysate and phage–lactoferrin treatments (except for strains no. 70 and 110) (Supplementary Materials, Table S1). The difference in CFU/mL between phage vB_SauM-C lysate and the phage–lactoferrin cocktail after 24 h of incubation was statistically significant in the case of strains 70 and 370. In the case of strains 113, 124, 203 and 352, no statistical significance was reported (Supplementary Materials, Table S2).

## 3. Discussion

Bacteriophages are an effective tool against biofilms formed by nosocomial, which are often multi-drug-resistant, strains of bacteria [20,21]. This has been proven by numerous studies by various research groups [8,22,23]. However, even though phages were successful where antibiotics have failed, they also seem to have their limitations [24,25,26]. Not all bacteriophages can penetrate to the inner layers of the biofilm and are only able to lyse the bacteria from the outermost layers. Additionally, extracellular polymeric substances secreted in biofilm formed by some bacterial genera can immobilize and inactivate the phages [27]. Furthermore, the emergence of phage-resistant bacteria has been reported [26,28]. Therefore, there is a need to find ways to counter these effects and increase the efficacy of phage therapy [26,27]. Currently, pairing bacteriophages and other antimicrobial agents such as antibiotics or essential oils are being investigated with promising results [14,29,30,31].

Antimicrobial proteins (AMPs), such as lactoferrin, are naturally occurring proteins that act as natural barriers against infection [32]. They are seen as another alternative to antibiotics in combat of antibiotic-resistant strains of bacteria. Lactoferrin has proven antimicrobial activity against various pathogens such as *Herpes simplex* [33], *Papillomavirus* [34], *Pseudomonas aeruginosa* [16], *Salmonella enterica*, *Streptococcus* sp. and *Staphylococcus* sp. [32,35,36].

We have observed that the use of lactoferrin alone had a moderate effect on *S. aureus* biofilm biomass and metabolism. The doses influencing biofilm metabolic state were 1.0 mg/mL and 10 mg/mL, and there was no statistically significant difference between those concentrations. However, the treatment of MDRSA biofilms with lactoferrin did not reduce the number of cells in the biofilm in a statistically significant way. It is therefore possible that lactoferrin acted as a bacteriostatic agent rather than a bactericidal one and prevented further biofilm formation. This was also observed by other research groups. Singh et al. [21] and Ammons et al. [37] have reported that the addition of lactoferrin to the medium prevented *Pseudomonas aeruginosa* from forming biofilms. Additionally, Quinteiri et al. [17] have observed that application of 2.5 mg/mL lactoferrin hydrolysate solution on biofilms attached to glass surfaces caused biofilm dispersion.

Since lactoferrin can influence biofilm dispersion in a significant way, it is therefore proposed to use it as an additive to other antimicrobials to increase their effectiveness. For example, it was reported that lactoferrin increases the inhibitory activity of penicillin up to 4-fold in penicillin-susceptible *S. aureus* strains and up to 16-fold in penicillin-resistant strains by reducing β-lactamase activity [38,39]. Similar results were observed when lactoferrin was paired with other antimicrobials and used against strains of *E. coli* [40], *P. aeruginosa* [37,41,42], *Candida* sp. [43] and *S. epidermilis* [44]. Furthermore, reports by Ammons et al. and Leitch and Willcox suggest that pairing lactoferrin with other compounds (that are not antibiotics) such as xylitol [37,42] or lysozyme [44] have resulted in increased antimicrobial effect. Taking this into account, the pairing of lactoferrin with phages seems to be a logical course of action. However, data on the use of the phage-lactoferrin mixture are very scarce. There are reports that the use of lactoferrin increased phage stability and tolerance to environmental factors [45,46,47], and there are few in vivo studies of phage–lactoferrin mixture’s effectiveness. Experiments performed by Zimecki et al. on mice models reported that a combination of lactoferrin (10 mg i.v.) and T4 phage reduced the bacterial load of *E. coli* in liver more effectively that each of the components separately [48]. Golshahi et al. [45] have observed that the use of lyophilized phages in lactose/lactoferrin in a ration of 60:40 improved phage performance when delivered as inhalable aerosol. However, there are not enough data to conclude whether the phage–lactoferrin mixture can be safely used and whether the use of such a cocktail will increase the effectiveness of the treatment. Therefore, in our work we have decided to analyze if the use of a phage–lactoferrin cocktail will result in increased effectiveness against biofilms formed by clinical strains of MDRSA [19]. Since we have observed that there was no significant difference in activity between 1.0 mg/mL and 10 mg/mL concentrations of lactoferrin, we have chosen to use the lower concentration for our studies. The phage concentration was chosen to be 10^9^ PFU/mL based on our previous reports [8]. We have observed that the use of a mixture resulted in significant decrease in all parameters: biomass, metabolic activity and CFU/mL of *S. aureus* biofilms after just 4 h of incubation. The effect of a mixture was more pronounced than the use of phages alone, with statistically significant differences after 12 h and 24 h of incubation. Furthermore, we have observed that the use of lactoferrin prolonged the activity of bacteriophages on the biofilm and prevented its re-growth; this was observed after 24 h of incubation if phages were used alone. This corresponds with the reports of other researchers, which suggests that lactoferrin boosts and prolongs the effects of other antimicrobials [44,45,47]. Therefore, it can be assumed that the use of the phage–lactoferrin cocktail has potential application against biofilms formed by multi-drug-resistant bacteria, though the detailed mechanism remains to be determined [15,16,47]. We believe that more in vitro studies involving other phage types and bacterial genera, followed by in vivo studies, i.e., on *Galleria mellonella* or *Caenorhabditis elegans* models, could deliver more detailed data on phage–lactoferrin synergy and effectivity, helping to answer the question if phage–AMPs mixtures can be used as one of the treatment methods of multi-drug-resistant infections.

## 4. Materials and Methods

### 4.1. Bacterial Strains

A total of 18 multi-drug-resistant *Staphylococcus aureus* clinical isolates were chosen from the collection of the Department of Medical Microbiology, the Medical University of Gdańsk. Strains were selected based on their biofilm forming ability and were previously described and characterized [18,19].

### 4.2. Bacteriophages

Bacteriophages vB_SauM-A, vB_SauM-C and vB_SauM-D were isolated from different wastewater treatment plants and were previously characterized [18]. Their anti-biofilm activity was analyzed and described [8]. Phage propagation, purification and enumeration were performed as described previously [18]. Final phage titer used in this study was 10^9^ PFU/mL.

### 4.3. Lactoferrin

Lactoferrin from bovine milk (Sigma-Aldrich, St. Louis, MO, USA) was dissolved in LB (Luria-Bertani) broth and filtered through 0.22 µm cellulose acetate filter (Merc, Darmstadt, Germany) to form a stock solution of 20 mg/mL and stored at 4 °C. Final concentrations used in the study were 10 mg/mL, 1 mg/mL and 0.1 mg/mL [16,32].

### 4.4. Lactoferrin Influence on Phage Activity

An amount of 100 µL of phage lysate with titer 10^9^ PFU/mL was mixed with 100 µL of lactoferrin at final concentration of 10 mg/mL and then incubated at 4 °C for 5 days. Every 24 h, a 10 µL sample was collected, serial dilutions were made and the mixture was titrated using double agar plate technique. The plates were incubated overnight at 37 °C and then scanned for plaques. The phage titer was calculated based on the number of plaques formed [18,49].

### 4.5. Assessment of Biofilm Biomass Using Crystal Violet Staining

Biofilms were grown on 96-well plates (Nest Biotechnology, Wuxi, China) in accordance with previously described protocols, with minor modifications [19]. Each well was inoculated with 200 µL of bacterial suspension, and the microtiter plates were incubated for 24 h at 37 °C. After incubation, established biofilms were washed with distilled H_2_O, and 200 µL of phage, lactoferrin or phage–lactoferrin mixture in LB was added to a set of wells. After an incubation period (4 h, 12 h or 24 h) at 37 °C, the wells were washed with distilled H_2_O, 100 µL of 1% crystal violet (Sigma Aldrich, St. Louis, MO, USA) solution was added to each well and the plate was incubated for 15 min at 37 °C. Excess stain was rinsed off by running tap water until the water was colorless, and the plate was left to air dry. To solubilize the dye bound to the biofilm, 200 µL of ethanol–acetic acid–water (30:30:40) was added to the wells, and the optical density at 595 nm was measured in the microplate spectrophotometer (BioTek Instruments, Winooski, VT, USA).

### 4.6. Assessment of Biofilm Metabolic Activity Using MTT

Biofilms were set and treated in accordance with protocol described above. After incubation period, MTT solution in PBS was added to final concentration of 0.5 mg/mL in 100 µL to each well and incubated at 37 °C for 1 h. After staining, the MTT solution was removed, and 200 mL of acidified isopropanol was added to dissolve the MTT formazan product. The absorbance was measured at 540 nm using a microplate spectrophotometer [19].

### 4.7. Enumeration of Cells in Biofilm Using CFU/mL Count

Biofilms were set and treated in accordance with protocol described at point 4.5. After the incubation period, the number of bacteria adhered to the surface of microplate wells was enumerated in accordance with previously described protocol. Therefore, 200 µL of 0.9% NaCl was added to each well, and biofilm cells were suspended by vigorous pipetting. The 10-fold serial dilutions were immediately performed in 0.9% NaCl and 40 μL of the dilutions were directly plated on LB plates.

### 4.8. Statistical Analysis

All the experiments were performed in triplicates that were averaged to produce means used for analysis. Mean values were compared using the t-test. Differences were considered statistically significant if *p* < 0.05.

## Data Availability

Raw data are available from the authors upon request.

## Supplementary Materials

The following supporting information can be downloaded at: https://www.mdpi.com/article/10.3390/antibiotics11091256/s1, Table S1: *p* values for statistical comparison between influence of phage vB_SauM-D and vB_SauM-D + 0.1 Lf on cell count (CFU/mL) of MDRSA biofilm, Table S2: *p* values for statistical comparison between influence of phage vB_SauM-C and vB_SauM-C + 0.1 Lf on cell count (CFU/mL) of MDRSA biofilm.
