# Supplementary material for: Activity of Phage–Lactoferrin Mixture against Multi Drug Resistant Staphylococcus aureus Biofilms
Source: Antibiotics (Basel). 2022 Sep 16;11(9):1256. doi: 10.3390/antibiotics11091256 (PMC9495459; doi:10.3390/antibiotics11091256)
Supplement: Supplementary file 1 [file antibiotics-11-01256-s001.zip › antibiotics-1917413-supplementary.pdf]

Table S1 P values for statistical comparison between influence of phage vB_SauM-D and vB_SauM-D+0.1 Lf on cell count (CFU/mL) of MDRSA biofilm

	MDRSA strain no.										
	44	70	110	113	115	124	203	316	317	352	370
**P value**	0,0042 **	0,0039 **	0,1456 ns	0,1689 ns	0,0011 **	0,0394 *	0,0170 *	0,0186 *	0,0173 *	0,0027 **	0,0042 **

Table S2 P values for statistical comparison between influence of phage vB_SauM-D and vB_SauM-D+0.1 Lf on cell count (CFU/mL) of MDRSA biofilm

	MDRSA strain no.					
	70	113	124	203	352	370
**P value**	0,0141 *	0,3173 ns	0,1662 ns	0,1998 ns	0,0547 ns	0,0257 *
